# Tracking the activity-dependent diffusion of synaptic proteins using restricted photoconversion of Dendra2

**DOI:** 10.3389/fncel.2015.00367

**Published:** 2015-09-22

**Authors:** Frédéric Cassé, Stéphane Martin

**Affiliations:** Centre National de la Recherche Scientifique UMR7275 – Laboratory of Excellence “Network for Innovation on Signal Transduction, Pathways in Life Sciences, ” Institut de Pharmacologie Moléculaire et Cellulaire, University of Nice – Sophia AntipolisValbonne, France

**Keywords:** synapse, live cell imaging, Dendra2, neuronal activity, SUMO

## Abstract

Spines are small protrusions on dendritic membranes receiving inputs from axonal termini. They consist in a head connected to the dendritic shaft by a narrow neck and contain multiple synaptic proteins that interact in a coordinated manner to allow for synaptic communication. This process involves many proteins that are moving in and out spines. However, comparing this synaptodendritic movement in basal and stimulated conditions is very challenging. Here we describe an elegant method to measure the activity-dependent diffusion of synaptic proteins using Dendra2 photoconversion. We provide a successful method to obtain Dendra2-photoconverted images and a step-by-step procedure to analyze the data. This live-imaging approach may also apply to investigate the diffusion of proteins across other subcellular compartments or organelles including but not restricted to, nucleus, nucleolus, ER, or vesicular structures. Once the imaging system is set up, data can be acquired in 1–30 min and analyzed in approximately 1–4 h.

## Introduction

Our view of neuronal communication has progressed enormously in the recent years. Indeed, the synapse is now seen as a very dynamic compartment where all processes are orchestrated both in time and space (Choquet and Triller, 2013). Among these processes is the continuous reorganization of the protein composition at synapses through the modulation of their interaction and/or diffusion in and out of the spine in an activity-dependent manner. This dynamic reorganization relies on protein-protein interactions that are often, if not always, regulated through post-translational modifications.

Important technical advances in live-cell imaging such as Fluorescent Recovery After Photobleaching (FRAP) and Fluorescence Loss In Photobleaching (FLIP) have given a better understanding of protein dynamics in various sub-neuronal compartments (Geva-Zatorsky et al., 2012). However, tracking, measuring, and comparing the diffusion properties of a specific protein of interest between the synaptic area and the dendritic shaft in basal and stimulated conditions is still quite challenging. Over the past years, several photoactivatable fluorescent proteins (PAFPs) have been described (Chudakov et al., 2007a; Gould et al., 2009) where their initial fluorescence could be rapidly switched by irradiation at a specific wavelength. Among them is the monomeric photoswitchable Dendra2 fluorescent protein that has been optimized for fast cellular maturation and for its efficient red photoconversion using UV activation at approximately 400 nm (Gurskaya et al., 2006; Chudakov et al., 2007a,b). After a UV-light irradiation, Dendra2 is irreversibly photoconverted to its red state and is highly photostable making this fluorescent tool extremely useful to precisely track intracellular protein movements in real time.

Dendra2-protein tagging has been previously used in the context of protein trafficking in neurons. For instance, photoswitchable fluorescent proteins have been used to track the protein Tau along axons (Zempel et al., 2013), to follow the actin dynamics in presynaptic termini (Flynn et al., 2009), or to visualize the subcellular distribution and real-time transport of the ionotropic ATP-gated P2X7 receptors in rat hippocampal neurons (Shrivastava et al., 2013). More recently, the expression of Dendra2-tagged proteins has been used to target mitochondria and to analyze their transport and morphology in sensory axons of living mice (Bolea et al., 2014). Despite such exciting advances in the field, these Dendra2 photoconversion studies were mostly performed on large neuronal areas thereby preventing the ability to assess the effect of cell activity on the dynamic properties of a defined protein within a specific subcellular compartment.

Here, we provide a step-by-step protocol to achieve an efficient photoconversion of a Dendra2-tagged protein in spines of a living neuron and to precisely track the synaptic diffusional exit of a photoconverted protein in real time. We also describe a detailed method to compare the properties of this transport in basal and stimulated conditions within the same spine. To this aim, we measured the basal and stimulated synapto-dendritic dynamics of the sumoylation enzyme Ubc9 fused N-terminally to Dendra2 in living rat hippocampal neurons (Dendra2-Ubc9).

Sumoylation is an essential post-translational modification (PTMs) by which the small ubiquitin-like modifying peptide SUMO is covalently attached to a lysine residue of a target protein (Matunis et al., 1996). SUMO conjugation is achieved via Ubc9, the sole SUMO-conjugating enzyme of the system. The role of sumoylation is always the modulation of the modified protein function. The molecular consequences of sumoylation are multiple. Sumoylation may lead to conformational changes, mask protein-protein interaction sites or alternatively create a new binding interface (Gwizdek et al., 2013; Henley et al., 2014). The proper functioning of such modifications requires a rigorous spatial and temporal control of the associated enzymatic machinery. Recently, using restricted GFP-photobleaching and Dendra2-photoconversion, we demonstrated that the activation of glutamatergic mGlu5 receptors (mGlu5R) transiently promotes the transient synaptic trapping of Ubc9 leading to an increased sumoylation in spines and consequently, to the modulation of neuronal excitability (Loriol et al., 2014).

Here we provide a comprehensive method to compare the diffusional properties of a protein of interest in basal and stimulated conditions. To achieve this aim, we followed the real time synaptic exit of a specific protein using restricted Dendra2-photoconversion. This detailed protocol also represents a powerful approach to compare the diffusional properties of specific proteins before and after a pharmacological stimulation on the same spine thereby allowing the direct assessment of the synaptic exit in activated conditions. Importantly, this Dendra2 photoconversion method can also apply to measure and compare the dynamic properties of proteins located in various subcellular compartments or organelles including, but not restricted to nuclei, nucleoli, growth cones, ER, or Golgi outposts as well as other vesicular structures, both in basal and stimulated conditions.

### Experimental design

**Figure 1:**
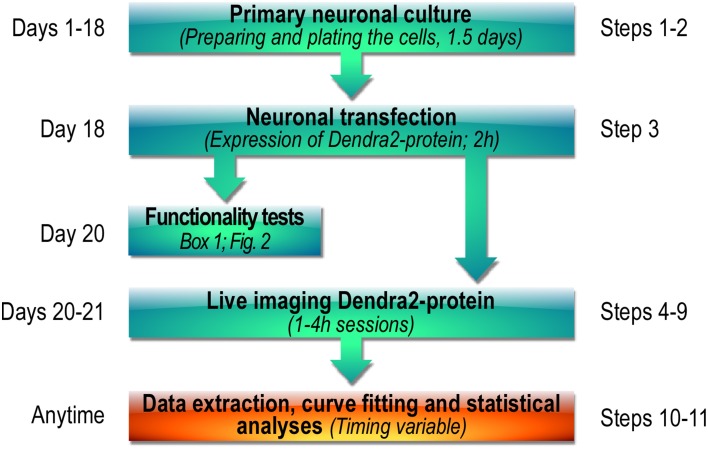
**Workflow diagram showing the main steps of the photoconversion protocol used to assess the activity-dependent synaptic transport of Dendra2-tagged proteins**.

Measuring the diffusional properties of synaptic proteins in living neurons is still quite challenging experimentally but is essential to assess the regulation of a specific target protein in basal and stimulated conditions. We designed a protocol that provides a basis to directly visualize and compare the synaptic exit of a tagged protein before and after cell activation. We fused the photoactivatable Dendra2 protein to the sole conjugating enzyme of the SUMO system, Ubc9 (Dendra2-Ubc9) in order to visualize in real time, the synaptic exit of this essential enzyme. We then achieved the photoconversion of Dendra2-Ubc9-expressing spines using the photoactivation unit of a spinning disk confocal microscope (UltraView Vox, Perkin Elmer). The photoactivation unit triggers the irradiation of a region of interest (ROI) thereby allowing the spatially restricted photoconversion of Dendra2 proteins. The main steps of our imaging protocol are depicted in Figure 1.

Importantly, the entire procedure can be adapted to any other confocal microscope equipped with a photoactivation unit and a temperature-controlled stage/chamber. However, it should be noted that in any case, the diffusion of the protein of interest has to be significantly slower than the maximum acquisition rate of the confocal microscope to allow the analysis of the process.

### Advantages and limitations of the approach

The current strategy can be adapted to any biological project where understanding the intracellular trafficking of a specific protein is of interest.

#### Advantages

- Adaptability to various confocal solutions bearing a photoactivation unit.
- Low cellular phototoxicity due to the combinaison of a spinning disk imaging solution with the spatially restricted photoconversion of Dendra2.
- Activity-dependent comparison of a dynamic process within a single spine/compartment.
- Successive synaptic photoconversion events may also permit the visualization of the direction and the measurement of the rate at which Dendra2-photoconverted molecules are transported along dendrites following their exit from the spine head.

#### Limitations

- Toxicity of the overexpressed Dendra2-tagged protein.
- Speed of the biological process *vs*. rate of the image acquisition.
- Number of neuronal culture dishes required to test the biological effect of a pharmacological treatment on spines.

## Materials (European catalog numbers)

### Reagents

– Neurobasal medium (Gibco, cat. no. 21103-049); Store at 4°C.
– HBSS medium (Lonza, cat. no. BE10-543F).
– B27 (50x; Gibco, cat. no. 17504-044).
– Glutamax 200 mM (100x; Gibco, cat. no. 35050-038).
– DiMethyl SulfOxide (DMSO; Sigma, cat. no. D8418)**! CAUTION** This chemical is corrosive (skin corrosion/burn, eye damage and corrosive) and toxic when inhaled, swallowed and on contact with the skin. Wear appropriate personal protective equipment and manipulate it under a hood.
– Antibiotic solution (100x), Penicillin-streptomycin (Pen/Strep; Contains 5000 units of Potassium Penicillin and 5000 μg Streptomycin Sulfate per ml; Lonza, cat. no. DE17-603E).
– Horse Serum (Dutsher, cat. no. 500105).
– L-Polylysine (Sigma, cat. no. P2636G).
– Trypsine-EDTA (Lonza, cat. no. BE17-161E).
– Trypan blue (Sigma, cat. no. T8154).
– HEPES (Sigma, cat. no. H3375).
– NaCl (vwr, cat. no. 27810.295).
– KCl (vwr, cat. no. 26764.260).
– CaCl_2_(Sigma, cat. no. 21114).
– MgCl_2_(vwr, cat. no. 25108.295).
– Tris Base (Euromedex, cat. no. 26-128-3094-B).
– Glucose (vwr, cat. no. 101175P).
– Lipofectamine 2000 transfection reagent (Invitrogen, cat. no. 52887).
– (S)-3,5-DiHydroxyPhenylGlycine (DHPG; Abcam, cat. no. 120007).
– Phorbol 12-Myristate 13-Acetate (PMA; Sigma, cat. no. P8139)**! CAUTION** This substance is very toxic when swallowed and on contact with the skin (carcinogen, mutagen, toxic for organs, and reproduction). Wear appropriate personal protective equipment.

### Reagent setup

– **Heat-inactivated horse serum (HS)** Inactive HS by heating it for 30 min at 56°C. Then, aliquots can be frozen at −20°C and stored for several months.
– **Plating medium** Supplement neurobasal medium with 2% B27, 5% HS, 2 mM glutamax, and 1% Pen/Strep.
– **Culture medium** Supplement neurobasal medium with 2% B27 and 1% Pen/Strep.
– **Transfection medium** Supplement neurobasal medium with 2% B27.
– **Earle's Buffer** Prepare Earle's buffer with the following final concentration: HEPES 25 mM, NaCl 140 mM, KCl 5 mM, CaCl_2_1.8 mM, MgCl_2_ 0.8 mM, Glucose 5 mM in ddH_2_O. Adjust buffer pH to 7.4 with a solution of non-titrated Tris Base 1 M.

### Equipment

– Microscope glass coverslips, round 24 mm, Thickness No.1 (vwr, cat. no. 631-0161).
– Cellstar® Cell Culture Dishes (Greiner Bio One, cat. no. 627-160).
– Heated insert Petri dish 35 mm (Leica, cat. No 11533095).
– Tweezers (Dumont, cat. No.4 Extra fine tips).
– Fine scissors (Roboz, cat. no. RS-5603).
– Binocular microscope (Leica, cat. no. MZ6).
– Hemocytometer Neubauer chamber (vwr, cat. no. 15170-173).
– Immersion Oil Type A (*Nd* = 1.515 at 23°C; Nikon, cat. no. MXA 20234).
– EM-CCD Camera (Hamamatsu, cat. no. C9100-02).
– Inverted fluorescence microscope (Nikon, cat. no. Eclipse Ti-E).
– Confocal scanner disk (Yokogawa, cat. No. CSUX1-A3).
– 100x Plan apochromat oil objective NA 1.4/ WD (Nikon).
– Quick exchange platform (Warner instruments, cat. no. QE-1).
– Single in-line solution heaters (Warner instruments, cat. no. SH-27B).
– Temperature controller (Warner instruments, cat. no. TC-344B).
– Photoactivation unit (PhotoKinesis device, Ultraview Vox, Perkin Elmer).
– Eight channel perfusion valve control system (Warner instruments, cat. no. VC-8).
– Data analysis software: Volocity 6.3 (Perkin Elmer); Image J; Prism 4 (GraphPad software, Inc).

### Construction of the Dendra2-Ubc9 expression vector

The Dendra2-Ubc9 expression construct used in our applications was made by inserting the Dendra2 coding sequence (Evrogen JSC, Russia) in frame with the N-terminus of Ubc9 with the Gateway recombination technology (Invitrogen). The Dendra2-Ubc9 construct has been entirely sequenced to check the integrity of the fusion protein. The Gateway destination vector for the fusion of the Open Reading Frame to the N-terminus of mouse Ubc9 sequence is a generous gift from Dr Niedenthal (Jakobs et al., 2007).

**Figure 2:**
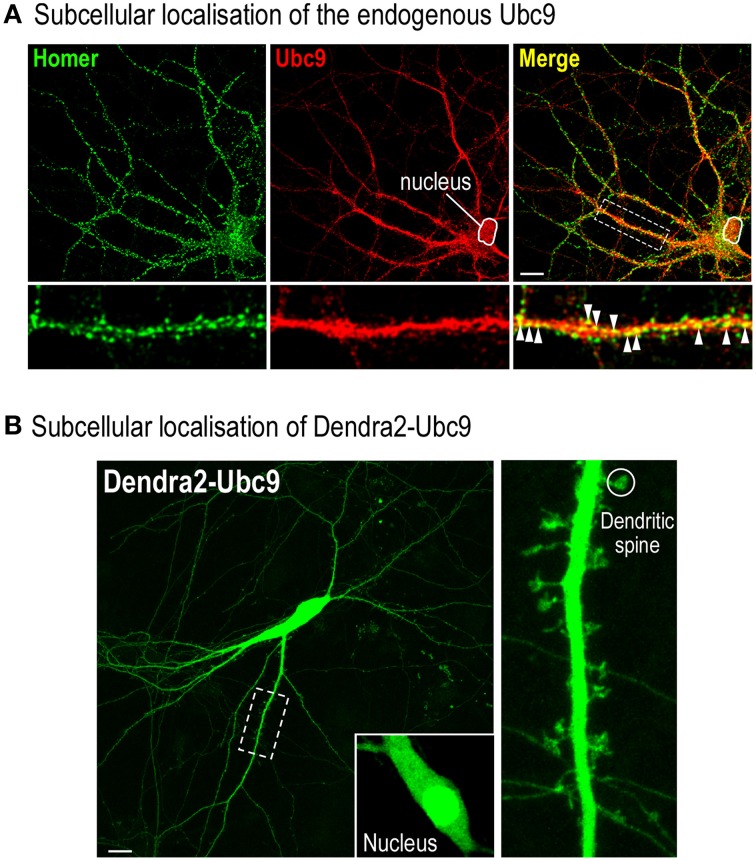
**Assessing the potential deleterious effect of the Dendra2 protein tagging. (A)** Representative distribution of the endogenously expressed Ubc9 in a 20 DIV rat hippocampal neuron. Fixed cells were permeabilised for 20 min at RT in PBS containing 0.1% Triton X100 and 10% Horse Serum (HS). Neurons were then immunolabelled with mouse anti-Ubc9 (1/50, BD Bioscience) and rabbit anti-Homer1 (1/200; Synaptic System, Germany) antibodies overnight at 4°C in PBS containing 0.05% Triton X100 and 5% HS. Cells were washed three times in PBS and incubated with the appropriate secondary antibodies conjugated to either Alexa488 (post-synaptic Homer staining) or Alexa594 (endogenous Ubc9) in PBS containing 5% HS with 0.05% Triton X100 for 1 h at RT. Merge color (yellow) shows the colocalisation between the proteins indicating that part of Ubc9 is expressed at Homer-positive post-synaptic sites (arrowheads). Note that, as expected, a significant proportion of Ubc9 immunoreactivity is present in the nucleus. Scale bar, 20 μm. **(B)** Representative image of a 20 DIV Dendra2-Ubc9-expressing rat hippocampal neuron. Note that Dendra2-Ubc9 is partly localized in the nucleus (inset) indicating that the Dendra2 tag does not impair its nuclear translocation. Dendra2-Ubc9 fluorescence is also distributed in dendrites and spines (circle). Scale bar, 20 μm.

*Note*: It is essential to verify the functionality of the tagged protein and to show that the selected tag does not impair the subcellular localization (Figure 2) and the overall activity of the studied protein (Loriol et al., 2014).

## Procedure

**Figure 3:**
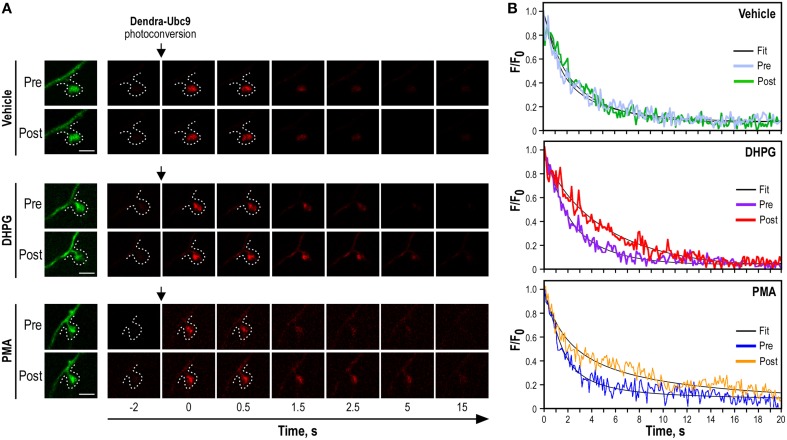
**Synaptic Dendra2-Ubc9 photoconversion experiments. (A)** Time-lapse series of confocal images of photoconverted Dendra2-Ubc9 red fluorescence in spines before and after pharmacological stimulations. Images of spine before Dendra2-Ubc9 photoconversion are shown in green (left). Following the synaptic photoconversion, neurons were incubated with the mGlu5R agonist DHPG (50 μM), with the PKC activator PMA (2 μm) or in control (vehicle) solution for 10 min at 37°C as indicated. Dendra2-Ubc9 from the same spine was then photoconverted a second time and imaged for 20 s. Scale bar, 1 μm. **(B)**. Representative sample paired recording traces of normalized fluorescence values obtained from individual photoconverted Dendra2-Ubc9 expressing spines before (Pre) and after (Post) vehicle, DHPG or PMA treatment as shown in **(A)**. The thin black curves represent the corresponding fits. Note that some parts of this figure derived from Loriol et al. (2014).

**Figure 4:**
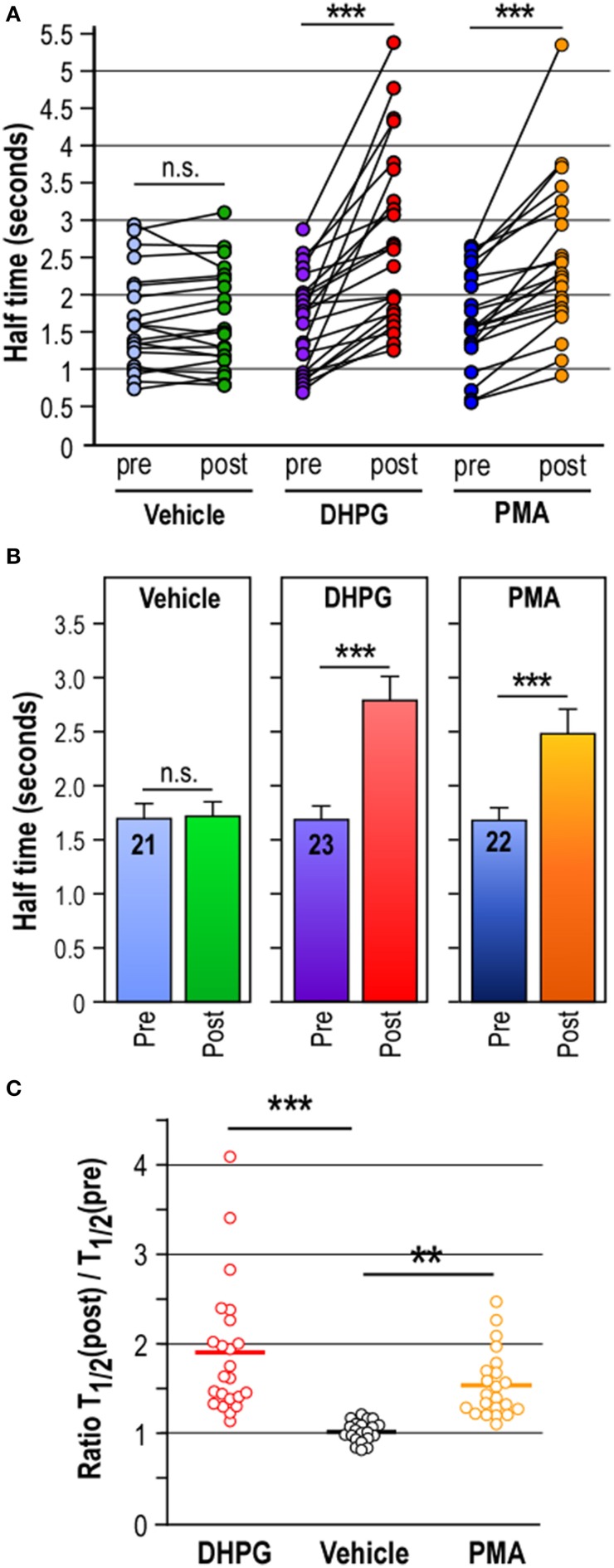
**Computational analyses of synaptic Dendra2-Ubc9 photoconversion data. (A)** Scatter plots of computed half-time of photoconverted Dendra2-Ubc9 fluorescence diffusion in spines pre- and post- pharmacological treatment. Paired *t*-test: Control Vehicle, *n* = 21; DHPG, *n* = 23; PMA, *n* = 22. n.s., not significant; ^***^P < 0.0001 compared with unstimulated (Pre) conditions. **(B)**. Histograms showing the mean ± s.e.m. of the fluorescence half-time constant in seconds calculated for the exponential decay fit from independent synaptic Dendra2-Ubc9 photoconversion experiments as shown in Figure 3B. Paired *t*-test: Control Vehicle, *n* = 21; DHPG, *n* = 23; PMA, *n* = 22. n.s., not significant; ^***^P < 0.0001 compared with unstimulated (Pre) conditions. **(C)**. Scatter plots showing the mean of half-time constant ratio of (Post/Pre) in control Vehicle (1.001 ± 0.025; *n* = 21), DHPG (1.824 ± 0.156; *n* = 23) and PMA (1.511 ± 0.078; *n* = 22) conditions. One-Way ANOVA were performed with a Newman-Keuls post-test for multiple comparison data sets. ^**^P < 0.01; ^***^P < 0.001. These data indicate that the activation of the PKC pathway is sufficient to transiently promote the diffusional trapping of Dendra2-Ubc9 in spines. Note that some parts of this figure derived from Loriol et al. (2014).

1. Preparing primary neuronal culture (Timing 1.5 d)
  This first section reports the step-by-step procedure to prepare primary hippocampal neurons from embryonic day 18 (E18) Wistar rats (Kaech and Banker, 2006; Loriol et al., 2013, 2014).
  - Sterilize 24 mm glass coverslips at 121°C for 1 h 30 in a glass beaker and prepare a coating solution containing 0.1 mg/ml of L-polylysine in sterile H_2_O. Place one glass coverslip per cell culture dish, add the coating solution on the top and incubate overnight at room temperature (RT). The next day, carefully wash the coverslips twice with sterile water under agitation, twice with HBSS at RT and finally incubate the coverslips in plating medium at 37°C in a 5% CO_2_ incubator until use.
    *Note*: L-polylysine is toxic to neurons. Therefore, extensive washes under mild agitation should be performed to completely remove the chemical (**Troubleshooting**, see Table 1).
  - Anesthetized a pregnant Wistar rat 18 days post-fertilization. Recover the E18 embryos in sterile HBSS medium at RT after euthanasia of the rat by decapitation.
    *Note*: All procedures are subjected to animal welfare regulations and must be validated by a national ethics committee. All our procedures were approved by the Animal Ethics Committee *(CIEPAL: Comité Institutionnel d'Éthique Pour l'Animal de Laboratoire N*°*28, Nice, France)* with the project reference NCE/2012-63.
    Extract the brain and remove the meninges with sterile forceps under a binocular microscope. Microdissect and collect all hippocampi in a 15 mL tube filled with HBSS at RT (Seibenhener and Wooten, 2012).
    *Note*: To avoid glial cell invasion and to get consistent and pure hippocampal culture, completely remove the meninges and ensure that all the cortical tissue and the white matter have been removed from the preparation (**Troubleshooting**, see Table 1).
  - Wash three times with HBSS at RT and incubate hippocampal tissue at 37°C for 15 min in 9 mL HBSS supplemented with 1 mL Trypsin-EDTA. Carefully wash the trypsinized hippocampi three times with the warm culture medium and gently dissociate the cells by triturating with a 1 mL-pipette tip until a homogeneous suspension is obtained.
    *Note*: Avoid creating air bubbles to ensure minimal cell damages during the trituration step.
  - Add 20 μl of the cell suspension to a 1.5 mL tube containing 180 μl of Trypan blue solution *(Dilution factor 10)*, homogenize the suspension by gently flicking the tube and wait 1–2 min at RT. Count the number of healthy unstained cells with hemocytometer *(dead cells are labeled in blue)*. The expected number of neurons recovered per hippocampus is ~200.000 cells. A higher number of cells (>300,000) per hippocampus indicates a contamination with cortical cells.
    Plate the neurons in plating medium at a density of 100,000 cells per coverslip and incubate at 37°C in a 5% CO_2_incubator. Replace the plating medium 3 h after seeding by the culture medium.
    *Note*: Always use pre-warmed (37°C) culture medium and do not let the coverslips to dry at any time to maintain cell viability.
2. Neuronal culture maintenance (Timing 18–19 d)
  Maintain neuronal culture in the incubator for 18–19 days and fed once a week with 200 μl of pre-warmed culture medium.
3. Transfection of hippocampal neurons (Timing 2 h)
  Transfect hippocampal neurons with the DNA of interest using lipofectamine 2000 following the manufacturer's instructions. First, pre-warmed neurobasal medium and prepare the transfection mix. In tube A, dilute 2 μg of DNA in neurobasal medium to a final volume of 100 μl. In tube B, place 97 μl plus 3 μl of lipofectamine 2000. After 5 min, combine both tubes, mix 2 s and incubate for 20 min at RT. Then, wash the neurons twice in neurobasal medium without antibiotics, add the transfection mix onto the cells and replace the dishes in the incubator (37°C, 5% CO_2_) for 1 h.
  *Note*: Keep the initial culture medium from each dish at 37°C in the incubator during the transfection steps.
  Rinse the transfected cells twice in pre-warmed neurobasal medium and incubate the neurons with their initial conditioned medium. Leave neurons in the incubator for 24–48 h before the imaging session depending on the level of protein expression you want to achieve (Karra and Dahm, 2010).
  *Note*: Pre-warm each solution to 37°C. Do not let the coverslips to dry at any time to maintain cell viability. Always perform the transfection steps using neurobasal medium ***without*** antibiotics, as it may severely damage the cells (**Troubleshooting**, see Table 1).
4. Imaging synaptic photoconverted Dendra2-Ubc9 (Timing 4 h)
  Use pre-warmed (37°C) Earle's buffer. Turn on all electronic equipments on the microscope and set the heated stage and the in-line solution heater to 37°C.
5. Calibration of the photoactivation unit
  The photoactivation unit must be aligned to allow the accurate photoconversion of the Dendra2-protein in spine. The manufacturer's instructions must be followed to precisely calibrate the photoactivation unit of the microscope setup and to ensure that the XYZ position is maintained during the imaging session.
  For Volocity users, select **Calibrate UltraVIEW PhotoKinesis Device** from the tool menu. Follow the wizard instructions and the calibration will occur automatically. For the fine calibration step, prepare a homogenous sample e.g., ink from a highlighter pen. Click the button **Next**, Volocity will bleach a specimen area and adjust accordingly. Select **Finish**.
6. Take the culture dish from the incubator. Carefully place the coverslip in a pre-heated imaging holder insert with tweezers, wash twice with pre-warmed Earle's buffer and place the cells on the heated stage of an inverted microscope with a 100x (1.4-NA) objective.
  *Note*: Ensure you work fast enough to minimize temperature changes, as the morphological aspect of neuronal spine is highly sensitive to temperature variations (Roelandse and Matus, 2004; **Troubleshooting**, see Table 1). If you do not have an incubator near the confocal microscope, move the cells carefully in a 37°C pre-warmed box.
7. Identify a spiny Dendra2-Ubc9-expressing hippocampal neuron (Figures 2, 3). Dendra2-Ubc9 is partly located within the nucleus and also distributed in dendrites and spines (Figure 2). Be sure to select spines of similar shape in you experiments since spine morphology may affect the diffusion rate of synaptic proteins (Simon et al., 2014).
  *Note*: Dendra2 excitation at 488 nm may induce its photoconversion so it is essential to use the minimum laser intensity necessary to visualize the Dendra2 signal (**Troubleshooting**, see Table 1).
8. Define the acquisition parameters
  We recommend selecting high magnification (100x) and high numerical aperture (NA = 1.4 or above) objectives since spines are small dendritic structures (~1 μm of diameter). The magnification of the objective lens should be chosen such that the effective pixel size of the sample is small enough to minimize localization errors due to pixel size (Betzig et al., 2006).
  The optimum temporal resolution will depend on the diffusion parameter of the studied protein. The acquisition frequency will be therefore set with respect to the dynamic properties of the sample. For instance, we imaged the synaptic exit of the photoconverted Dendra2-Ubc9 at 10 Hz.
  A major advantage of a spinning disk solution is the higher acquisition rate together with a reduced exposure of the sample to laser illumination, which concurrently leads to a lower degree of photobleaching and minimizes cellular phototoxic effects. Indeed, photobleaching and phototoxicity in live cell imaging are essentially induced by excited fluorochrome, which generate reactive oxygen species (ROS; Dixit and Cyr, 2003). These ROS react with various cellular components such as lipids and nucleic acids and lead to a loss of fluorescence and to cellular toxicity (Dobrucki et al., 2007).
  Acquisition parameters i.e., laser power and exposure time depend on the confocal microscope setup used, the power and age of the laser and the type of detectors used. To image the green non-photoconverted Dendra2 fluorescence, set the 488-nm excitation laser power and exposure time to the lowest possible intensity (e.g., laser power < 1% and exposure time of less than 100 ms) to avoid unwanted Dendra2 photoconversion.
  To visualize the red photoconverted Dendra2 protein, select the cell body of a neuron and photoconvert Dendra2-Ubc9 using the 405-nm laser line. Then, adjust the 561 nm excitation laser line and exposure time until you visualize a red fluorescence signal. For instance, we set the 561-nm excitation laser power to 20% with an exposure time of 75 ms to image the red-photoconverted Dendra2-Ubc9 signal.
  *Note*: After 1 min, the red-photoconverted Dendra2-Ubc9 fluorescence from the cell body is evenly diffused in the neuron. Select a ROI on a dendrite and measure the red fluorescence over a 30s period. Define the photobleaching rate (*R*) by computing the ratio between the red fluorescence intensity value within the selected ROI at the end of the recording (*F*) with the intensity value measured initially (*F*_0_):
  $$R\text{ = }F\text{/}F_{0}$$
  The *R*-value must be close to 1, if not decrease 561-nm laser line power and/or exposure time.
9. Imaging Ubc9-Dendra2 photoconversion
  Find another Dendra2-Ubc9-expressing hippocampal neuron and check that the red fluorescence signal before the photoconversion event is close to zero. Since time-lapse imaging generates files of very large size, crop to image the dendrite and the spine of interest in order to reduce file size. Select a ROI of 1 μm in diameter on the selected spines. Live-image for 5–10 s before the spine photoconversion to get the mean red fluorescence intensity over time. Then, photoconvert the synaptic Dendra2-Ubc9 molecules using the 405-nm laser line at its maximum intensity for the shorter possible period of time and live image the red fluorescence channel at high frequency to allow the analysis of the synaptic exit of the protein of interest. In our case, we used a 30 ms photoconversion period and imaged the red-photoconverted Dendra2-Ubc9 at 10 Hz for 20s after the photoconversion (Figure 3).
  *Note*: The photoconversion trigger has to be significantly shorter than the synaptic exit time of the target protein and the acquisition frequency must be set to ensure that the synaptic exit of the photoconverted target protein is clearly assessable (**Troubleshooting**, see Table 1).
  Once acquired, use the perfusion system to deliver Earle's buffer containing either the drug of interest or its corresponding control vehicle solution (1 mL/min) and incubate for the time indicated by the manufacturer at 37°C to achieve the biological effect. Paired recordings of synaptic Dendra2-protein photoconversion are used to assess the effect of a specific drug on the synaptic exit of the protein of interest. Therefore, perform a second photoconversion of the selected spine after the pharmacological incubation and image as above (Figure 3). For control conditions, perform the second synaptic photoconversion after incubation in Earle's buffer containing the vehicle.
10. Data analysis of Dendra2-Ubc9 diffusion (Timing variable)
  The saved imaging files can be open with a dedicated imaging software or Image J with the LOCI Bio-formats importer plug-in. Select the ROI corresponding to the selected spine. First, measure the mean red fluorescence value before photoconversion to have a background value. Then, measure the red fluorescence from the photoconverted Dendra2-Ubc9 over time and export the raw data in a spreadsheet application like Microsoft Excel. Subtract the mean background value to red fluorescence intensity for each time point to obtain the red fluorescence intensity value (*F*) over the time course of the experiment. Calculate the variation of fluorescence over time to the initial red photoconverted fluorescence (*F*/*F*_0_); *F*_0_ representing the maximum intensity measured in the red channel right after the photoconversion event. Finally, trace the *F*/*F*_0_ curves as a function of time (Figure 3B).
  To compute the half-time of photoconverted Dendra2-Ubc9 fluorescence diffusion (*T*_1/2_), first export the *F*/*F*_0_ data as a function of time in Prism 4 (GraphPad software, Inc). Then, use analyzes prism4's tool menu, select non-linear regression (curve fit) and compute a one phase exponential decay to obtain the half-time constant. The equation is as follow:
  $$Y=\text{Span}\times {\text{e}}^{\text{ − }(\text{K}.\text{X})}\;+\text{ Plateau}$$
  *Y* is the *F*/*F*_0_ value; Plateau is the *Y*-value at infinite time; K is the rate constant, expressed in reciprocal of the X-axis time units. The half-time is in the time units of the X axis and is computed as ln(2)/K. To compare stimulated to unstimulated control conditions, calculate the ratio of half-time constant for each experiment by dividing the *T*_1/2_ obtained after treatment by the control *T*_1/2_ [Ratio = *T*_1/2_ (post-treatment)/*T*_1/2_ (pre-treatment); Figure 4].
11. Statistics analysis (Timing Variable)
  The data obtained can then be analyzed using Prism 4 (GraphPad software, Inc). To statistically compare the Dendra2-Ubc9 *T*_1/2_ results between control and a drug-treated condition on the same spine, use a *paired t*-test. The use of *t*-tests is only possible when the analyzed data follow a normal distribution and provide a *p*-value. A *p* > 0.05 may either reflect that there is no significant difference between two conditions or that the number of experiments performed is too low.
  Use unpaired *t*-test to compare the ratio of half-time constants of one treatment to another *(vehicle compared to DHPG for instance)*. Importantly, use One-Way ANOVA tests to statistically compare more than two groups together.
  First, compare the half-time of the red fluorescence decrease from two successive photoconversion events *on the same spine* by using the paired recording *t*-test. The number of points in each data set *(pre- and post-stimulation)* must be equal and organized in pairs *(one pair equivalent to one spine)*. In unstimulated condition, the unpaired *t*-test comparison of half-time value before and after photoconversion is not significant (*n* = 21) meaning that two consecutive synaptic Dendra2-Ubc9 photoactivations do not affect the exit rate of the protein in unstimulated basal condition (Figure 4A). In DHPG- or PMA-stimulated conditions, the paired *t*-test comparison of half-time values before and after the drug stimulation gives *p* < 0.0001 (DHPG, *n* = 23; PMA, *n* = 22) indicating that both DHPG and PMA treatments affect significantly the exit rate of Dendra2-Ubc9 from spines (Figure 4B).
  To statistically evaluate the effect of multiple pharmacological treatments, use a One-Way ANOVA test with a Newman-Keuls or a Bonferroni post-test for multiple comparison data sets to compare the pre- and post-stimulation ratio of half-time constants between vehicle and the different drug-treated conditions (Figure 4C). The statistical comparison of half-time ratio *(unstimulated vs. drug-stimulated)* gives significant *p*-values ranging from *p* < 0.05 to *p* < 0.001 (ANOVA; Vehicle vs. DHPG, *p* < 0.001; Vehicle vs. PMA, *p* < 0.01; PMA vs. DHPG, *p* < 0.05) meaning that activation of mGlu5R or PKC downstream pathways significantly affects the synaptic exit rate of Dendra2-Ubc9 (Figure 4C).

**Table 1:** **Troubleshooting table**.

**Problem**	**Possible reason**	**Solution**
**STEP 1**		
Glial cell invasion	Meninges and/or white matter contamination	Make sure that meninges and white matter are completely removed
Neuronal cell death	Presence of L-polylysine	Increase the number of washes after the coating step
	Low cell density	Increase the number of cells per dish Choose a different lot of horse serum
**STEP 3**		
No transfected neurons	Traces of antibiotic during transfection	Increase the number of washes before the transfection
**STEP 6**		
Absence of spines	Neuronal cultures are not mature	Make sure neuronal cultures are at least 18 DIV when performing such experiments
	Coverslips stay to long outside of the medium	Quickly mount the coverslip in the holder before the imaging
**STEP 6**		
Swollen dendrites	Neurons dried in the early stage	Ensure the coverslip does not dry at any time
**STEP 7**		
Neurons are red before photoconversion	The 488 nm light intensity is too high	Decrease the 488 nm laser light intensity
**STEP 9**		
Photoconversion outside the ROI and/or bleached the adjacent shaft area	Calibration is not set properly	Repeat the calibration process
	The photoconverted region is too large	Reduce the size of photoconversion ROI
	Movements of the sample	Make sure the insert remains steady throughout the imaging session
	The Focus stabilizing device is not on	Switch the Focus stabilizing device on

### Timing

Step 1: Preparation of primary neuronal culture 1.5d.
Step 2: Neuronal culture maintenance ~18–19 d.
Step 3: Transfection of hippocampal neurons 2 h + 1–2 d.
Steps 4–9: Dendra2-Ubc9 photoconversion imaging session 4 h.
Steps 10–11: Data and statistical analyses 1–3 h.

## Anticipated results

Our procedure describes a quantitative approach to measure and analyze the diffusion of proteins from a dendritic spine using two successive photoconversion of a Dendra2-tagged protein in basal unstimulated or stimulated conditions. Typical photoconversion experiments using a 405 nm-excitation laser line to photoconvert Dendra2-Ubc9 are shown in Figure 3. Using time-lapse microscopy, we provide a way to directly assess the exit of a specific protein by measuring the loss of the red photoconverted Dendra2-Ubc9 fluorescence from the spine in basal condition (Figures 3, 4). The amount of red-photoconverted fluorescence decreases over time in the spine head indicating that Dendra2-Ubc9 molecules move out of the spine.

In basal conditions, the half-time of the red fluorescence decrease from two successive photoconversion events on the same spine is not significantly different (n.s., paired *t*-test; *n* = 21 spines, Pre, *T*_1/2_ = 1.68 ± 0.15 s; Post vehicle, *T*_1/2_ = 1.70 ± 0.15 s; Figures 4A,B). This essential observation confirms that consecutive photoactivation events do not affect the exit rate of synaptic Dendra2-Ubc9.

The pharmacological stimulation of metabotropic glutamate mGlu5 receptors in hippocampal neurons with DHPG (50 μM, 10 min) before the second Dendra2-Ubc9 photoconversion leads to a significant delay in the exit rate of the red photoconverted fluorescence from the spine head (*n* = 23 spines, pre-DHPG, *T*_1/2_ = 1.63 ± 0.16 s; post-DHPG, 2.78 ± 0.26 s, ^***^p < 0.0001 paired *t*-test; Figures 4A,B). We also show here that the direct activation of the Protein Kinase C (PKC), a downstream kinase of the mGlu5R activation cascade, with PMA (2 μM PMA, 10 min) also leads to the significant decrease in the half-time exit of the synaptic photoconverted Dendra2-Ubc9 (*n* = 22 spines, pre-PMA, 1.69 ± 0.14 s; post-PMA, 2.46 ± 0.21 s; ^***^p < 0.0001 paired *t*-test; Figures 4A,B) indicating that the synapto-dendritic transport of Ubc9 is regulated by neuronal activity (Figure 4C; Loriol et al., 2014).

Thus, our protocol can be used in combination with classical FRAP experiments, to precisely assess and compare the diffusion properties of cytosolic proteins in and out of a target spine in basal and stimulated conditions. Interestingly, repeated Dendra2 photoconversion events on the same spine could also be used to directly assess the direction and the speed of the photoconverted protein transport along dendrites after their synaptic exit. In addition, our “paired” Dendra2-approach can be extended to other subcellular compartments to investigate the activity-dependent regulation of a variety of intracellular proteins.

## Author contributions

FC and SM designed the protocol, performed experiments, prepared the figures and wrote the manuscript.

### Conflict of interest statement

The authors declare that the research was conducted in the absence of any commercial or financial relationships that could be construed as a potential conflict of interest.
